# Changes in Surface Soil Organic Carbon Fractions and Their Pool Management Indices Along an Altitudinal Gradient in Karst Mountains in Relation to the Expansion Degrees of *Chimonobambusa utilis*

**DOI:** 10.3390/biology15010025

**Published:** 2025-12-23

**Authors:** Long Tong, Qingping Zeng, Lijie Chen, Xiaoying Zeng, Ling Shen, Fengling Gan, Minglan Liang, Lixia Chen, Xiaoyan Zhang, Lianghua Qi

**Affiliations:** 1 International Centre for Bamboo and Rattan, Beijing 100102, China; tonglongcq@outlook.com; 2 Chongqing Academy of Forestry, Chongqing 400036, China; zqping0802@163.com (Q.Z.); chlj314@163.com (L.C.); xyy1538000593@163.com (X.Z.); 13983302857@163.com (L.S.); 3 Chongqing Key Laboratory of Forest Ecological Restoration and Utilization in the Three Gorges Reservoir Area, Chongqing 400036, China; 4 Chongqing Key Laboratory of Surface Process and Ecological Restoration in the Three Gorges Reservoir Area/Karst Research Team, Chongqing Key Laboratory of Carbon Cycle and Carbon Regulation of Mountain Ecosystem, School of Geography and Tourism Science, Chongqing Normal University, Chongqing 401331, China; ganfengling@cqnu.edu.cn; 5 Nanchuan District Forestry Industry Development Service Center, Chongqing 408499, China; m18234323932@163.com; 6 Chongqing Jinfo National Nature Reserve Management Affairs Center, Chongqing 408400, China; clx20150115@126.com; 7 Jinfo Mountain Forest Farm, Nanchuan District, Chongqing 408400, China; 13880104965@163.com

**Keywords:** high-altitude karst regions, soil carbon sequestration capacity, expansion of *Chimonobambusa utilis*, bamboo forest ecosystems

## Abstract

Soil organic carbon fractions and pool management indices are critical for the ecosystem function of bamboo forests; however, their response to varying degrees of expansion of *Chimonobambusa utilis* (EDCU) and altitudinal gradients remains poorly understood in high-altitude karst regions. In this study, 225 samples (three replicate soil samples, each with five duplicate samples) were collected from 45 typical soil sites in the Jinfo high-altitude karst mountains, China. This study investigated the effects of three EDCUs (low, moderate, and high expansion) and five altitudinal gradients (1300–1500 m, 1500–1700 m, 1700–900 m, 1900–2100 m, and 2100–2300 m) on root elemental composition, soil properties, soil organic fractions, and pool management indices. The results revealed that root total C, N, R_C:P_, and R_N:P_ decreased with increasing altitude, whereas root total C, N, P, and R_C:N_ also increased significantly with increasing EDCU. Compared with those at low and moderate EDCU, the POC:SOC (34.12%), HFOC (32.73 g kg^−1^), and HFOC:SOC (37.07%) ratios were highest at high EDCU along the altitudinal gradient of 1700–1900 m. Meanwhile, the L (2.38), LI (2.01), and CMI (174.55) ratios reached their highest values at moderate expansion degrees of *Chimonobambusa utilis* within the altitudinal gradient of 1900–2100 m. Moreover, redundancy discriminant analysis (RDA) and structural equation modeling (SEM) revealed that the soil carbon pool management index was significantly positively associated with soil properties through direct pathways and negatively correlated with root elemental composition through indirect pathways. In general, the quality of the carbon pool in *Chimonobambusa utilis* is optimal within the moderate expansion degrees of *Chimonobambusa utilis* within the altitudinal gradient of 1900–2100 m. The findings of this study establish a theoretical basis for the expansion of *Chimonobambusa utilis* in high-altitude karst regions and provide scientific evidence to support the increase in the carbon sequestration capacity of bamboo forest ecosystems in these mountainous areas.

## Simple Summary

This study investigated the effects of the degrees of expansion of *Chimonobambusa utilis* (EDCU) and altitudinal gradients on root elemental composition, soil properties, soil organic fractions, and pool management indices. The results demonstrated in this study showed the effects of expansion degrees of *Chimonobambusa utilis* and altitudal gradients on root elemental composition, soil properties, soil organic carbon fractions, and carbon pool management indices. The results showed that the carbon pool management index was positively correlated with soil properties and carbon fractions but negatively correlated with root elemental composition. Overall, reducing limitations on root-mediated organic carbon inputs along altitudinal gradients may enhance the adaptability of *Chimonobambusa utilis* to nutrient-poor environments. Moreover, soil carbon pool quality reached optimal levels during moderate expansion phases within the 1900–2100 m altitudinal gradient. These findings provide a theoretical foundation for understanding the carbon sequestration potential of *Chimonobambusa utilis* expansion in high-altitude karst regions. Soil enzyme activity and stoichiometric ratios are indirectly influenced by soil bulk density and root characteristics. Overall, both carbon (C) and phosphorus (P) limitations were observed at the lowest MRB level (<20%), whereas higher MRB levels exhibited P limitation exclusively, with no significant C limitation. These findings provide a scientific basis for promoting green and sustainable management practices in bamboo-invaded broad-leaved forest ecosystems.

## 1. Introduction

With global climate change and increasing bamboo forest invasion, evergreen broad-leaved forests are being progressively replaced by bamboo forests, leading to the establishment of bamboo-dominated ecosystems [1,2]. Recent studies have demonstrated that bamboo forest invasion not only alters the community structure and landscape patterns of broad-leaved forests but also significantly affects soil physicochemical properties and microbial activity, thereby disrupting the biogeochemical cycles of these ecosystems. Specifically, bamboo forest invasion impacts the storage and stability of the soil carbon pool in broad-leaved forests by altering the composition and spatial distribution of soil organic carbon (SOC), consequently influencing key ecosystem functions such as carbon sequestration and carbon emissions. Therefore, a comprehensive and in-depth analysis of the ecological impact mechanisms of bamboo forest expansion on SOC pool dynamics is essential for effectively managing bamboo forest encroachment and safeguarding forest soil carbon dynamics under global climate change.

SOC is a crucial component of the forest carbon pool and is significantly influenced by factors such as forest succession and management practices [3,4,5]. In recent years, to gain a more comprehensive understanding of the complexity and stability of SOC, SOC has been categorized into labile and inert forms on the basis of the degree of decomposition and cycling rate. For example, SOC can be classified into particulate organic carbon (POC) and mineral-associated soil organic carbon (MOC) on the basis of particle size through physical methods or into light fraction organic carbon (LFOC) and heavy fraction soil organic carbon (HFOC) according to density via chemical methods [6,7,8]. Among them, the distribution ratios of labile SOC (e.g., POC and LFOC) can indicate soil quality by reflecting microbial decomposition, SOC mineralization, and subtle carbon pool changes, which characterize SOC turnover rates and respond sensitively to bamboo expansion [9]. In contrast, inert SOC (e.g., MOC and HFOC) forms when plant exudates and microbial residues bind with soil minerals, resulting in slow turnover and high resistance to microbial degradation, thus ensuring long-term stability. The labile and inert SOC contents significantly differ in terms of source, turnover, and functionality, which are often used as indicators of the SOC pool management index (CPMI) in forest ecosystems, particularly during the process of bamboo invasion [10,11]. Thus, studies that assess different expansion degrees of bamboo forests in evergreen broad-leaved forests under specific geological environmental conditions are needed to quantify the impacts of such practices on SOC fractions and pool management.

Recent advances emphasize the CPMI as a sensitive indicator for assessing changes in soil carbon dynamics within terrestrial ecosystems, highlighting its close association with the labile fraction of soil organic carbon and its significant influence on root elemental composition through soil properties [12,13]. For example, some recent studies have further demonstrated that the contribution of underground root systems to the CPMI is at least as significant as that of aboveground litter [14,15,16]. Fine root turnover contributes approximately 14% to 27% of the global net primary production and plays a crucial role in the CPMI [17]. Owing to the high lignin content and low levels of soluble organic nitrogen in fine root litter, its decomposition leads to an 8% increase in the CPMI, independent of the influence of root secretions [18]. Consequently, below-ground plant residue C plays a more substantial role in the formation of inert SOC (MOC and HFOC) than do above-ground residues [19,20]. However, most existing studies have focused predominantly on fallen leaves on the ground, which may overlook the critical role of root elemental composition within a specific topographic environment, that drives the SOC fractions and their pool management, which are strongly influenced by soil properties.

As a crucial topographic factor, the altitudinal gradient can create environmental gradients within regional ecosystems by influencing variables such as light intensity, temperature regimes, precipitation patterns, and soil nutrient conditions, thereby exerting both direct and indirect effects on the SOC fraction and its pool management [21,22]. Differences in soil water content and thermal conditions caused by varying altitudinal gradients can influence microbial activity, thereby regulating litter decomposition rates and affecting plant root growth and development, ultimately impacting labile SOC (such as POC and LFOC) and CPMI [23,24]. Some studies have indicated that the labile SOC and CPMI have a unimodal distribution along an altitudinal gradient, with the highest values occurring at mid-altitudes [25]. In light of this, a more comprehensive understanding of the SOC fraction and its pool management under specific geological environments requires an in-depth investigation of the elemental composition of bamboo roots in broad-leaved forests. This research gap limits a comprehensive understanding of the relationship between fine root decomposition and the carbon cycle in bamboo forest ecosystems at different altitudes.

*Chimonobambusa utilis*, known as the “crown of bamboo” in China for its prominent autumn shoots, spreads efficiently via underground rhizomes and gradually invades adjacent broad-leaved forests [26]. Owing to the strict “no interference” conservation policy in China, which prohibits the removal or disturbance of any plant species within the karst area of the UNESCO World Heritage Site in China, unrestricted expansion of *Chimonobambusa utilis* has taken place [27]. This uncontrolled growth has intensified interspecific competition between *Chimonobambusa utilis* and other native plant species, thereby significantly altering the SOC fraction and pool management, which are driven by extensive karstification, shallow surface soils, severe rocky desertification, and an inherently fragile ecological environment [28]. In contrast, current research on invasive bamboo species has focused predominantly on common bamboo and moso bamboo in nonkarst regions, as well as shrub species in forested ecosystems [29,30]. Thus, the following two hypotheses should be present: (1) the expansion of *Chimonobambusa utilis* along an altitudinal gradient significantly changes the SOC fraction and its pool management index; and (2) during the changes in the SOC fraction and its pool management index driven by the expansion of *Chimonobambusa utilis* along an altitudinal gradient, the root characteristics and soil properties exhibit significant variation and play a prominent role in karst high-altitude mountainous regions.

To verify the above two hypotheses, the present study selected typical high-altitude karst regions with the expansion of *Chimonobambusa utilis* in Southwest China as the study area. The three expansion degrees of *Chimonobambusa utilis* (low, moderate, and high expansion) and five altitudinal gradients (1300–1500 m, 1500–1700 m, 1700–1900 m, 1900–2100 m, and 2100–2300 m) were selected as research objects. The following studies were conducted: (1) the impacts of the expansion degrees of *Chimonobambusa utilis* and altitudinal gradient on root elemental composition and soil physicochemical properties; (2) the responses of SOC fractions, stocks, and pool management indices to the expansion degrees of *Chimonobambusa utilis* via the altitudinal gradient; and (3) the relationships between the SOC pool management index and root and soil properties under varying altitudinal gradients and the expansion degrees of *Chimonobambusa utilis.* This study aims to provide essential insights into the role of bamboo forests in global carbon cycling and to support the optimization of bamboo forest management strategies and policy decisions in high-altitude karst regions of China.

## 2. Materials and Methods

### 2.1. Site Description

The Jinfo National Nature Reserve is located in the southern part of the Nanchuan District, at the southeastern edge of the Chongqing Municipality, close to the tri-junction area of Sichuan, Guizhou, and Chongqing (106°54′–107°27′ E, 28°46′–29°38′ N). The region lies along the axis of a broad, gentle syncline and features typical limestone karst landforms. The upper part consists of Permian Qixia Formation limestone, with extensive gentle slopes, well-developed karst depressions, and large caves. The middle section is composed of Silurian shale and siltstone, whereas the lower part contains Cambrian and Ordovician limestone and dolomite, showing small and microkarst features, exposed travertine deposits, and complex karst morphology. The average annual temperature at higher altitudes is 8.2 °C, with an average annual precipitation of 1434.5 mm. In contrast, at lower altitudes, the average annual temperature is 16.6 °C, and the average annual precipitation is 1286.5 mm. Owing to the significant elevation range, the soil distribution from the mountain base to the summit follows the sequence of yellow soil, dark brown soil, yellow–brown soil, and brown soil. Moreover, this area is characterized by small bamboo forests dominated by *Chimonobambusa utilis*, which are distributed mainly at altitudes ranging from 1300 to 2300 m. These forests serve not only as important plant resources but also as crucial sources of income for local communities. Under ongoing human activities, the distribution of *Chimonobambusa utilis* has expanded in the Jinfo Karst Mountains. It colonizes forest gaps created by anthropogenic disturbances and is widely present beneath broad-leaved forests, where it has become the dominant or even the sole understory species. As a result of long-term invasion by *Chimonobambusa utilis*, a distinct transitional zone has formed in Chengdu, making the area a representative site for studying the impacts of bamboo forest invasion on forest carbon cycling [13,31].

### 2.2. Experimental Design

**Figure 1:**
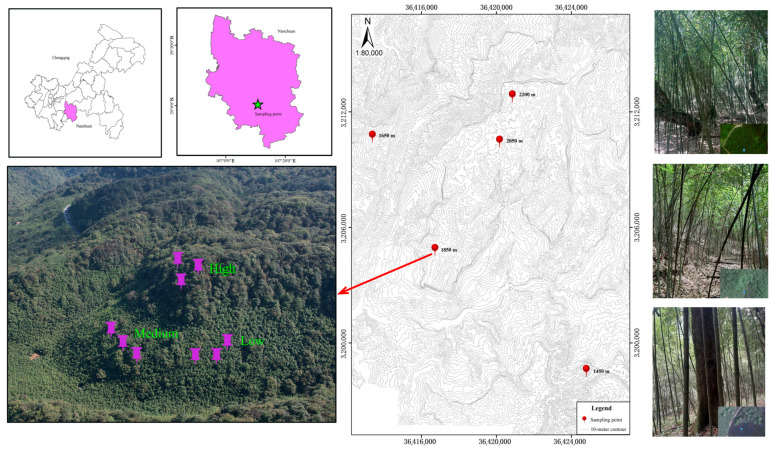
Sampling sites along an altitudinal gradient within bamboo forest expansion areas in high-altitude karst regions.

In mid-August 2024, the experimental site was located within the core natural distribution area of *Chimonobambusa utilis* in the Jinfo Karst Mountain region, which also constitutes a major production area for its shoots [32]. This research was conducted with the formal approval and support of the local forestry authorities and the relevant project administration. Field investigations revealed that human interference in the experimental area occurred mainly during the *Chimonobambusa utilis* shoot harvesting season in late August. To minimize such interference, all experimental plots were established and enclosed prior to the annual shoot season, thereby preventing human disturbance within the *Chimonobambusa utilis* forest on Jinfo Karst Mountain [33]. According to the Technical Regulations for Forest Resources Planning in China and field investigations conducted on *Chimonobambusa* within the Jinfo Karst Mountains, the optimal altitude range for species growth was determined to be between 1300 and 2300 m. This range was subsequently categorized into five 200 m altitudinal gradients: 1300–1500 m, 1500–1700 m, 1700–1900 m, 1900–2100 m, and 2100–2300 m. The altitudes selected for this study fell within the natural altitude range of the *Chimonobambusa utilis* distribution in Jinfo Karst Mountain, without exceeding its lowest or highest observed altitudes. In addition, the mixing ratio of bamboo and broad-leaved forests within the sample plots was determined on the basis of their relative dominance, as indicated by the comparison of the cross-sectional area at breast height between the two forest types. Within five altitudinal gradient ranges, continuous plots were established along the expansion degree front where *Chimonobambusa utilis* forest encroached upon evergreen broad-leaved forest, which were categorized into three expansion degrees on the basis of *Chimonobambusa utilis* forest coverage: low expansion (*Chimonobambusa utilis* proportion less than 20%), moderate expansion (*Chimonobambusa utilis* from 40% to 60%), and high expansion (*Chimonobambusa utilis* proportion greater than 80%). Each plot measured 20 m × 20 m, and the distance between the three expansion belts was maintained at 5 m. This spatial design complies with international standards for soil sampling in mountainous regions characterized by dense vegetation, such as bamboo and coniferous forests. These guidelines recommend maintaining a distance of no more than 5.0 m between internal replicates to ensure plot homogeneity while accounting for the inherent ecological heterogeneity associated with karst environments (Figure 1).

Furthermore, the plots used for root system sampling were identical to those designated for soil sampling. Root sampling of Fuzhuang bamboo in the surface layer (0–20 cm) was conducted concurrently with soil collection. Three medium-sized and rapidly growing *Chimonobambusa utilis* forests were selected as standard plots for the sampling of various types of fine roots on the basis of preliminary plot surveys. Fine roots, defined as root segments with a diameter of ≤2 mm, were collected from the 0–20 cm soil layer beneath the selected standard bamboo stands. In each plot, the five-point sampling method was employed to establish five sampling points. At each point, soil samples were collected via a stainless steel core sampler with dimensions of 10 cm in inner diameter and 10 cm in height [34]. Specifically, two sampling points were randomly selected within a 0.5 m radius from the trunk of each standard stand. After surface vegetation, such as weeds and fallen leaves, the fine roots were collected following the lateral root distribution patterns of *Chimonobambusa utilis*. The fine root samples collected from the same sampling points were subsequently thoroughly mixed and placed in sealed bags for low-temperature storage. These samples were then oven-dried at 70 °C until a constant weight was reached, ground via a mill, and passed through a 100-mesh sieve for the determination of total carbon (TC), total nitrogen (TN), and total phosphorus (TP) contents in the fine roots. Additionally, five sampling points were randomly selected in each plot via an “S”-shaped pattern, and soil samples from the 0–20 cm depth were collected via the five-point sampling method. Therefore, a total of 225 soil samples were collected, corresponding to the following sampling design: 5 altitudinal gradients × 3 expansion degrees of *Chimonobambusa utilis* × 3 plot replicates × 5 sampling points per plot. Each soil sample was thoroughly mixed and placed in self-sealing bags after visible coarse roots and stones were removed. The soil samples were subsequently air-dried and sieved through a 2 mm mesh to analyze their physicochemical properties as well as relevant carbon components [35].

### 2.3. Root and Soil Sample Measurements

The TC and TN contents in the fine roots and soil samples were analyzed via a CHNS/O elemental analyzer (Vario EL III, Rhein-Main-Gebiet, Germany). The TP content was determined via a continuous flow analyzer (SAN++, SKALAR, Breda, The Netherlands) after H_2_SO_4_–HClO_4_ digestion, and the stoichiometric ratios of TC, TN, and TP in both the soil and fine roots were then calculated [24,36].

The soil bulk density was determined via the 100 cm^3^ ring knife method (diameter: 5.05 cm, height: 5 cm), which is widely recommended for karst regions because of its ability to balance sample integrity with representativeness. Given the high gravel content and structurally fragmented nature of karst soils, coarse fragments exceeding 2 mm were removed prior to the initial bulk density calculation. A correction formula was subsequently applied to adjust the measured values by accounting for the mass and volume of the excluded material, thereby minimizing potential bias arising from the removal of coarse debris. To address the pronounced spatial heterogeneity characteristic of karst soils, five replicate measurements were performed in each sampling plot. The soil pH was measured with a portable pH meter (Sartorius PB-10, Göttingen, Germany) at a water-to-soil ratio of 2.5:1 (*v*/*v*). The soil particle size distribution was analyzed via laser diffraction using a Beckman Coulter LS13 320 laser particle size analyzer (Brea, CA, USA). The soil sediments were classified into clay, silt, and sand fractions on the basis of the established particle size ranges, and the relative proportions of each fraction were calculated [3,37].

### 2.4. Soil Organic Carbon Fractions

The total soil organic carbon (SOC) content was measured through high-temperature combustion via a TOC analyzer (Multi N/C 2100S, Jena, Germany). On the basis of differences in soil particle size, SOC can be physically separated into two fractions: particulate organic carbon (POC) and mineral-associated organic carbon (MOC). Following the methods of Camenzind et al. (2023) [3], sodium hexametaphosphate was applied for oscillatory dispersion. The suspension was then filtered and rinsed repeatedly with distilled water. The fraction retained on the sieve was defined as particulate organic matter, which was dried at 65 °C to a constant weight. The POC was subsequently determined via a TOC analyzer. The MOC content was derived by subtracting the particulate organic carbon value from the SOC content.

Additionally, SOC can be divided into two components on the basis of density: light fraction organic carbon (LFOC) and heavy fraction organic carbon (HFOC). The separation of LFOC and HFOC was carried out by oscillation and dispersion in a NaI solution. Following centrifugation and filtration of the suspension, the light fraction retained on the microporous filter membrane was first rinsed with CaCl_2_ solution and subsequently with distilled water. The residue was then dried at 65 °C to a constant weight, after which the LFOC content in the soil was determined via a TOC analyzer. The HFOC content was calculated as the difference between total SOC and LFOC [38].

### 2.5. Soil Organic Carbon Stock and Pool Management Index

The SOC stock in the surface soil layer was calculated as follows:

(1) $${\mathrm{SOC}}_{S}=\sum_{i=1}^{n}C_{i}\rho_{i}T_{i}\left(1-\theta_{i}\right)\times 10^{-1}$$

where ${\mathrm{SOC}}_{S}$ represents the SOC stock at a specific depth (t·hm^−1^); $C_{i}$ denotes the SOC content of the i-th soil layer (g·kg^−1^); $\rho_{i}$ represents the soil bulk density of the i-th soil layer (g·cm^−3^); $T_{i}$ refers to the soil thickness of the i-th soil layer (cm); $\theta_{i}$ is the volume percentage of gravel content larger than 2 mm in the i-th soil layer, which is used to ensure accurate estimation of carbon storage in the surface soil layer of rocky karst ecosystems (%); and n indicates the total number of soil layers included in the calculation. In this study area, the gravel content exceeding 2 mm in diameter was found to be negligible and therefore was not included in the analysis [15,39].

The soil carbon monoxide management index (CPMI) is defined as the ratio of the SOC content to the SOC content of a control soil, multiplied by the SOC activity index [10,40]. The CPMI serves as an effective indicator for evaluating changes in SOC induced by different soil management practices. As a systematic and sensitive monitoring tool, it accurately captures dynamic variations in SOC content and comprehensively reflects how external factors influence both the quantity and quality of SOC.

L = Ca/(SOC − Ca)(2)

LI = L/AO(3)

CPI = SOC/SOC_O_ (4)

CPMI = CPI × LI × 100(5)

where L represents the soil organic carbon pool activity; Ca denotes the content of active soil organic carbon, such as particulate organic carbon (POC) and light fraction organic carbon (LFOC); SOC refers to the total soil organic carbon content; LI indicates the carbon pool activity index; Ao represents the soil activity of the reference sample; SOCo denotes the total organic carbon content of the reference soil; CPI stands for the carbon pool index; and CPMI refers to the carbon pool management index. In this study, soil from an evergreen broad-leaved forest at an altitude of 400 m was selected as the reference soil [31,41].

### 2.6. Statistical Analysis

This study employed one-way ANOVA to analyze the significant differences in root elemental composition, soil physicochemical properties, carbon fractions, and the carbon pool management index across different altitudinal gradients and levels of expansion of *Chimonobambusa utilis*, followed by multiple comparisons via the least significant difference (LSD) method. Redundancy analysis (RDA) and the Mantel test were employed to investigate the relationships among the soil carbon fractions, carbon pool quality, and environmental factors. Additionally, a Monte Carlo permutation test was conducted to determine the significant environmental factors influencing the soil carbon fractions and carbon pool quality. All the statistical analyses were conducted via SPSS 25.0. Redundancy analysis, Mantel testing, and data visualization were performed with the aid of the vegan, dplyr, linkET, and ggplot2 packages in R 4.3.1 software. Structural equation modeling was employed to explore the influence pathways of root elemental composition and soil physicochemical properties on soil organic carbon fractions and carbon pool quality [2,42]. In addition, Shapiro-Wilk tests for normality (α = 0.05; *p* > 0.05 indicates adherence to a normal distribution; otherwise, outliers were examined or data transformation was considered) and Levene’s test for homogeneity of variance (α = 0.05; *p* > 0.05 indicates homoscedasticity; otherwise, data transformation was applied, or nonparametric alternatives such as Welch ANOVA or the Kruskal-Wallis H test were employed) were conducted on all experimental data. Parametric analyses were performed when both assumptions were satisfied. When violations occurred, the data were subjected to transformation and re-evaluated for assumption compliance; if assumptions remained unmet, nonparametric methods were utilized. This analytical framework adheres to established statistical guidelines and accounts for the inherent heterogeneity characteristic of karst habitat datasets, thereby ensuring the reliability and scientific rigor of the results [43].

## 3. Results

### 3.1. Changes in Root Elemental Composition and Stoichiometry

**Figure 2:**
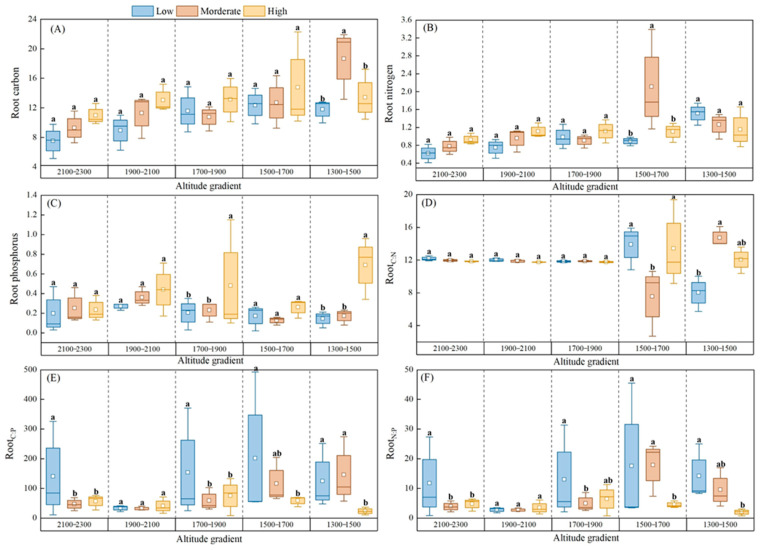
Altitudinal gradient of root elemental composition and stoichiometry at different expansion degrees of *Chimonobambusa utilis* in the high-altitude karst mountainous area. Note: A total of 225 soil samples were collected across 5 altitudinal gradients, with 3 bamboo expansion forest plots per altitude, each containing 3 replicate plots and 5 soil samples per plot. Indicators for each forest area were based on composite samples from 3 replicate plots (5 soil samples each) to reduce sampling variability. Error bars show the standard error; different lowercase letters above bars indicate significant differences among altitudes (*p* < 0.05, one-way ANOVA with Tukey’s test). (**A**) Root carbon; (**B**) Root nitrogen; (**C**) Root phosphorus; (**D**) Root C:N ratio; (**E**) Root C:P ratio; (**F**) Root N:P ratio. Different lowercase letters indicate significant differences between different patches of the same soil depth (*p* < 0.05) the same applies below.

The root elemental composition and stoichiometry varied significantly, and the altitudinal gradient as well as the expansion degree of *Chimonobambusa utilis* followed either a linear or unimodal pattern. Across the three expansion degrees of *Chimonobambusa utilis*, the average root total C, N, and P contents ranged from 10.43 to 12.72, from 0.95 to 1.20, and from 0.20 to 0.42 g·kg^−1^, respectively. In addition to the root total N and P contents, the root total C content increased significantly with decreasing altitude (*p* < 0.05; Figure 2). Moreover, the root total C and P contents increased significantly with increasing expansion degree of *Chimonobambusa utilis*, irrespective of the altitudinal gradient.

The R_C:N_, R_C:P_, and R_N:P_ ratios ranged from 11.28–12.07, from 36.05–65.00, and from 2.94–6.76, respectively, with respect to the expansion degrees of *Chimonobambusa utilis* (Figure 2). Across the three expansion degrees of *Chimonobambusa utilis*, root nutrient stoichiometry exhibited a unimodal relationship with altitude. The R_C:N_ ratio first decreased and subsequently increased along the atitudinal gradient, whereas the R_C:P_ and R_N:P_ ratios exhibited the opposite trend (Figure 2). Specifically, the R_C:N_ ratio reached its highest value at moderate expansion degrees of *Chimonobambusa utilis* and its lowest value at high expansion degrees of *Chimonobambusa utilis*, with the R_C:P_ and R_N:P_ ratios showing the reverse pattern.

### 3.2. Changes in Soil Physicochemical Properties and Stoichiometry

**Figure 3:**
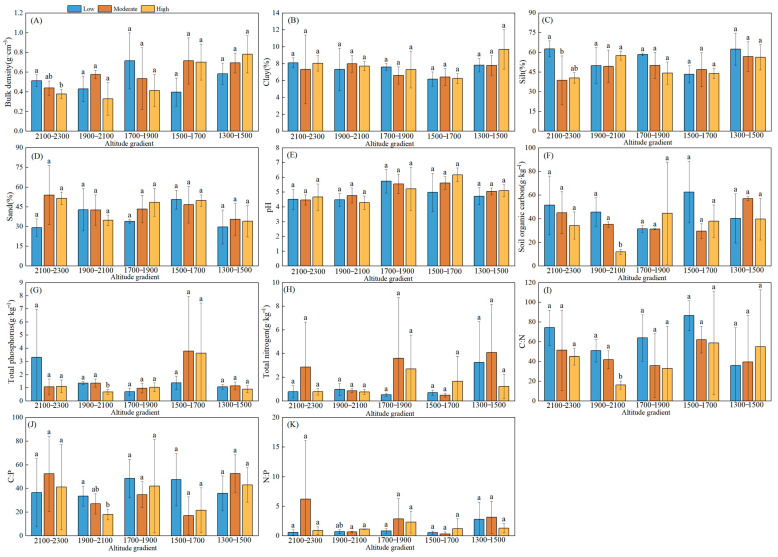
Altitudinal gradient of soil physicochemical properties at different expansion degrees of *Chimonobambusa utilis* in high-altitude karst mountainous areas. Note: A total of 225 soil samples were collected across 5 altitudinal gradients, with 3 bamboo expansion forest plots per altitude, each containing 3 replicate plots and 5 soil samples per plot. Indicators for each forest area were based on composite samples from 3 replicate plots (5 soil samples each) to reduce sampling variability. Error bars show the standard error; different lowercase letters above bars indicate significant differences among altitudes (*p* < 0.05, one-way ANOVA with Tukey’s test). (**A**) Bulk density; (**B**) Clay; (**C**) Slit; (**D**) Sand; (**E**) pH; (**F**) Soil organic carbon; (**G**) Total phosphorus; (**H**) Total nitrogen; (**I**) C:N ratio; (**J**) C:P ratio; (**K**) N:P ratio. Different lowercase letters indicate significant differences between different patches of the same soil depth (*p* < 0.05) the same applies below.

The results revealed that the altitudinal gradient and expansion degrees of *Chimonobambusa utilis* significantly affected the soil physicochemical properties (Figure 3). The soil BD tended to increase with decreasing altitude, and the BD values under moderate expansion degrees of *Chimonobambusa utilis* were significantly greater than those under other expansion degrees. The soil in this area had pH values ranging from 4.30 to 6.17, reflecting a decrease in the high expansion degrees of *Chimonobambusa utilis* observed in soils at altitudes of 1900–2100 m to lower levels in soils at altitudes of 1500–1700 m. The soil particle size composition was dominated by silt, followed by clay, with the sand content being the lowest. Both the clay and silt contents reached their maximum values within the altitude range of 1300–1500 m, whereas the minimum values were recorded at 1500–1700 m. In contrast, the sand particle content displayed an inverse trend. The TC and TN contents initially declined sharply, reaching minimum values at altitudes ranging from 1900–2100 m. They subsequently gradually increased and reached their maximum levels within the lowest altitude range of 1300–1500 m, irrespective of the expansion degree of *Chimonobambusa utilis*. Moreover, at altitudes ranging from 1900–2100 m, the TP and TC contents reached their minimum values, with a high expansion degree of *Chimonobambusa utilis*. Regardless of the altitudinal gradient, TP and TN reached their maximum concentrations at moderate expansion degrees of *Chimonobambusa utilis*. The C:N and C:P ratios decreased with increasing expansion degree of *Chimonobambusa utilis*, whereas the C:P and N:P ratios reached their maximum values at an altitude of 1300–1500 m, regardless of the expansion degree of *Chimonobambusa utilis*.

### 3.3. Changes in Soil Organic Carbon Fractions

**Figure 4:**
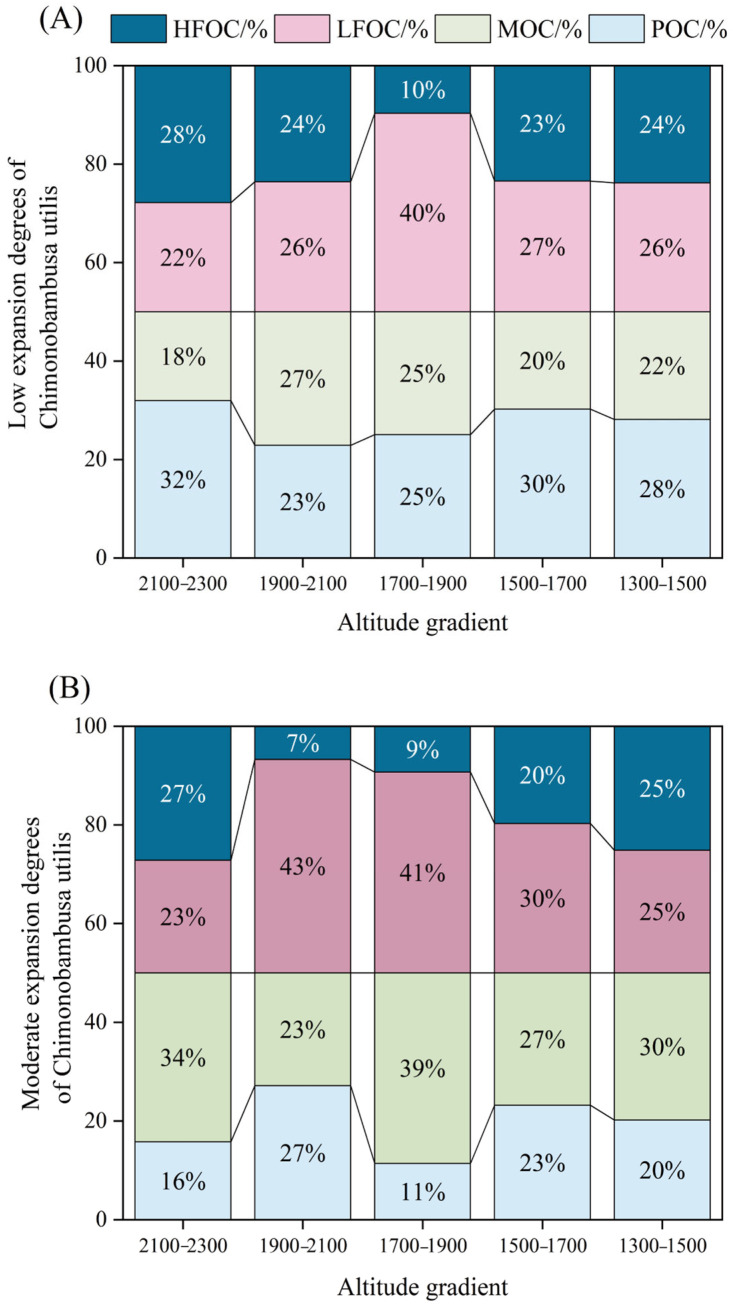

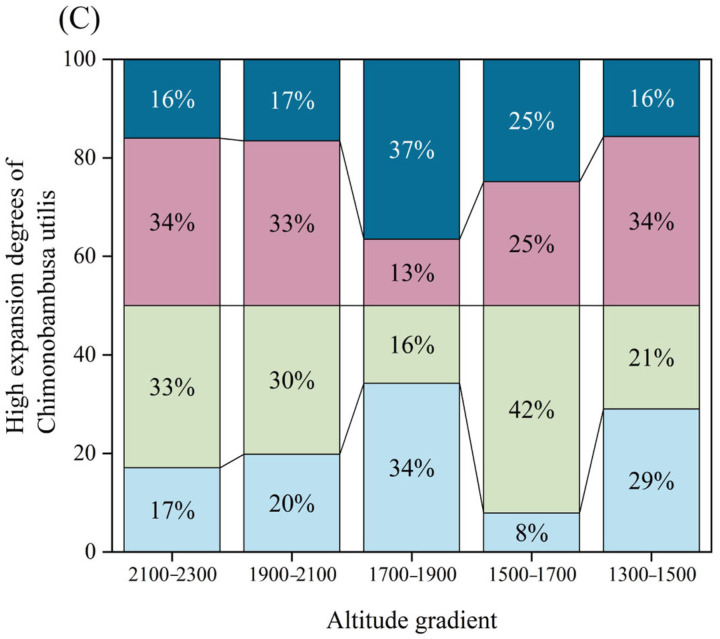
Altitudinal gradient of soil organic carbon fractions and their contributions to SOC at different expansion degrees of *Chimonobambusa utilis* in the high-altitude karst mountainous area. Note: A total of 225 soil samples were collected across 5 altitudinal gradients, with 3 bamboo expansion forest plots per altitude, each containing 3 replicate plots and 5 soil samples per plot. Indicators for each forest area were based on composite samples from 3 replicate plots (5 soil samples each) to reduce sampling variability. Error bars show the standard error (*p* < 0.05, one-way ANOVA with Tukey’s test). (**A**) At low expansion degrees of *Chimonobambusa utilis*; (**B**) At moderate expansion degrees of *Chimonobambusa utilis*; (**C**) At high expansion degrees of *Chimonobambusa utilis*.

The changes in soil organic carbon fractions and their contribution rates to soil total organic carbon with different expansion degrees of *Chimonobambusa utilis* and altitudinal gradients are shown in Figure 4. For example, the contents of POC and LFOC were highest in the altitudinal gradient of 1300–1500 m and lowest in the high expansion degrees of *Chimonobambusa utilis* within the altitudinal gradient of 1900–2100 m. Furthermore, along the altitudinal gradients from 1900 to 2300 m, the POC:SOC ratio increased with increasing altitude, whereas the MOC:SOC ratio decreased. This pattern indicates that increasing altitude promotes the relative accumulation of POC while concurrently reducing the formation efficiency of MOC, leading to divergent trends in their respective contributions to SOC across the gradient. Within any given elevation gradient, POC:SOC reached its maximum under low expansion degrees of *Chimonobambusa utilis* and its minimum under moderate expansion degrees, whereas MOC:SOC displayed a contrasting pattern. Under low expansion conditions, high organic input and favorable preservation promote POC accumulation, whereas transformation into MOC is restricted; in contrast, under moderate expansion, reduced plant-derived inputs and increased POC decomposition result in decreased POC stocks, but microbial-mediated processes facilitate more efficient stabilization of MOC. The LFOC and HFOC contents were lowest at altitudes ranging from 1900–2100 m and then gradually increased with increasing altitude. Moreover, the HFOC content and ratios of HFOC:SOC were significantly highest at altitudes ranging from 2100–2300 m and lowest at altitudes ranging from 1900–2100 m, whereas the LFOC:SOC ratios exhibited the opposite trend. Additionally, within the altitudinal gradient of 1700 to 1900 m, the POC:SOC (34.12%), HFOC (32.73 g kg^−1^), and HFOC:SOC (37.07%) ratios were highest at high expansion degrees of *Chimonobambusa utilis*. Moreover, the LFOC:SOC ratio was highest, whereas the HFOC:SOC ratio was lowest at moderate expansion degrees of *Chimonobambusa utilis*.

### 3.4. Changes in the Soil Organic Carbon Stock and Pool Management Index

**Figure 5:**
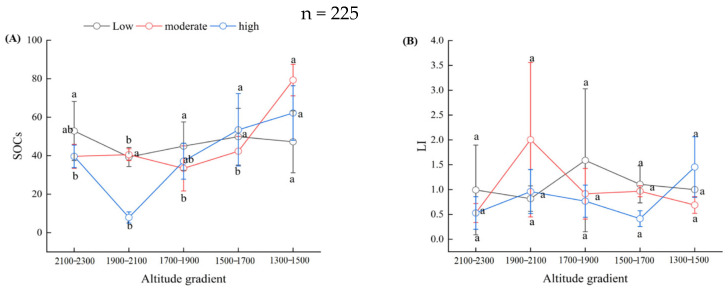

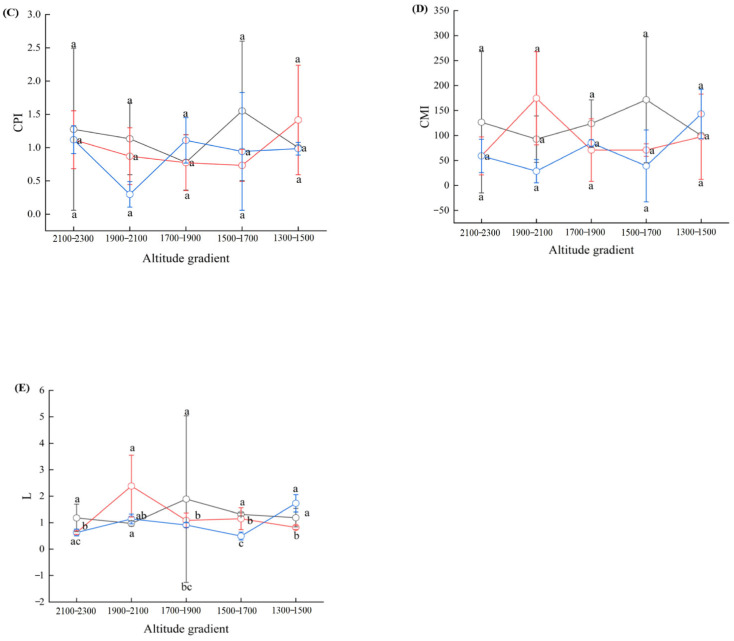
Altitudinal gradients of the soil organic carbon stock and pool management index at different expansion degrees of *Chimonobambusa utilis* in the high-altitude karst mountainous areas. Note: A total of 225 soil samples were collected across 5 altitudinal gradients, with 3 bamboo expansion forest plots per altitude, each containing 3 replicate plots and 5 soil samples per plot. Indicators for each forest area were based on composite samples from 3 replicate plots (5 soil samples each) to reduce sampling variability. Error bars show the standard error; different lowercase letters above bars indicate significant differences among altitudes (*p* < 0.05, one-way ANOVA with Tukey’s test). (**A**) SOCs: soil organic carbon stock; (**B**) LI: carbon pool activity index; (**C**) CPI: carbon pool index; (**D**) CPMI: carbon pool management index; (**E**) L: soil organic carbon pool activity.

The expansion degrees of *Chimonobambusa utilis* affected the soil organic carbon (SOC) stock and carbon pool management index (CMI) in high-altitude karst mountainous areas (Figure 5). Except for the altitudinal gradient of 2100–2300 m, the SOC stock and CPI increased as the altitudinal gradient decreased from 2300 m to 1300 m, with increases of 31.58% and 15.63%, 65.98% and 40.22%, and 115.04% and 47.66%, respectively, compared with the SOC stock in the altitudinal gradient of 1900–2100 m, regardless of the expansion degree of *Chimonobambusa utilis*. Furthermore, we found that there was no significant difference in the SOC stock between the low and moderate expansion degrees of *Chimonobambusa utilis*, with the highest value observed in the high expansion degree of *Chimonobambusa utilis*. The highest altitudinal gradient of 2100–2300 m presented the lowest values of L, LI, and CMI, whereas the altitudinal gradient of 1900–2100 m presented the highest values of L and LI. Furthermore, irrespective of the altitudinal gradient, the values of L, LI, CPI, and CMI exhibited a decreasing trend with increasing expansion degrees of *Chimonobambusa utilis*. In particular, for the altitudinal gradient of 1900–2100 m, the values of L, LI, and CMI were significantly highest at moderate expansion degrees of *Chimonobambusa utilis*.

### 3.5. Relationships Between Soil Carbon Fractions, Pool Management Index, and Environmental Factors

**Figure 6:**
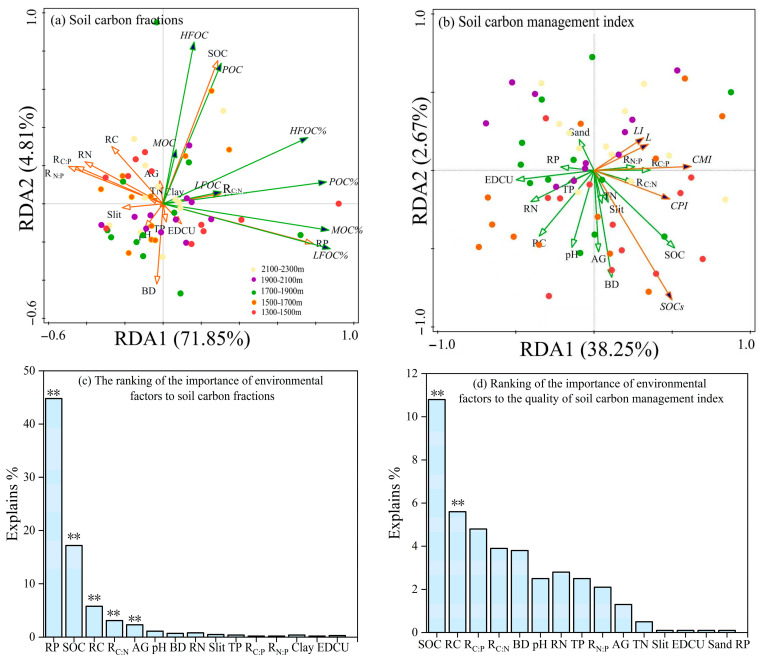
Results of redundancy discriminant analysis (RDA) illustrating the relative influence of soil carbon fractions (**a**,**b**) and the soil management index (**c**,**d**) in relation to environmental factors. Note: A total of 225 soil samples were collected across 5 altitudinal gradients, with 3 bamboo expansion forest plots per altitude, each containing 3 replicate plots and 5 soil samples per plot. Indicators for each forest area were based on composite samples from 3 replicate plots (5 soil samples each) to reduce sampling variability. Error bars show the standard error (*p* < 0.05, one-way ANOVA with Tukey’s test). The symbol “**” denotes a statistically highly significant correlation between the two elements (*p* < 0.01). (**a**) Soil carbon fractions; (**b**) Soil carbon management index; (**c**) The ranking of the importance of environmental factors to soil carbon fractions; (**d**) The ranking of the importance of environmental factors to the quality of soil carbon management index.

According to redundancy analysis, soil environmental factors across different altitudinal gradients have significantly distinct effects on soil organic carbon fractions and pool management indices. The RDA1 axis explained 71.85% and 38.25% of the variation in the soil carbon fractions and pool quality, respectively, whereas RDA2 accounted for 4.81% and 2.67%, respectively (Figure 6). This suggests that the RDA1 axis predominantly captures the variability in both soil carbon fractions and pool quality. Furthermore, the SOC, TN, and clay contents were significantly positively correlated with the organic carbon fractions, whereas BD exhibited a significant negative correlation with these fractions. AG showed a significant positive association with MOC. RP was identified as the most influential factor in determining soil carbon fractions, accounting for approximately 45% of the explained variation. SOC and RC demonstrated moderate explanatory power, whereas clay and EDCU made minimal contributions to the model’s explanatory capacity. With respect to the soil carbon pool, Silt and RC were significantly positively correlated with the soil carbon pool quality, whereas pH and BD were significantly negatively correlated. Moreover, SOC is the most influential factor in determining soil carbon pool quality, accounting for approximately 11% of the explained variation. R_C:P_ and R_C:N_ are secondary contributors, whereas Silt, EDCU, Sand, and RP exhibit the lowest explanatory power.

**Figure 7:**
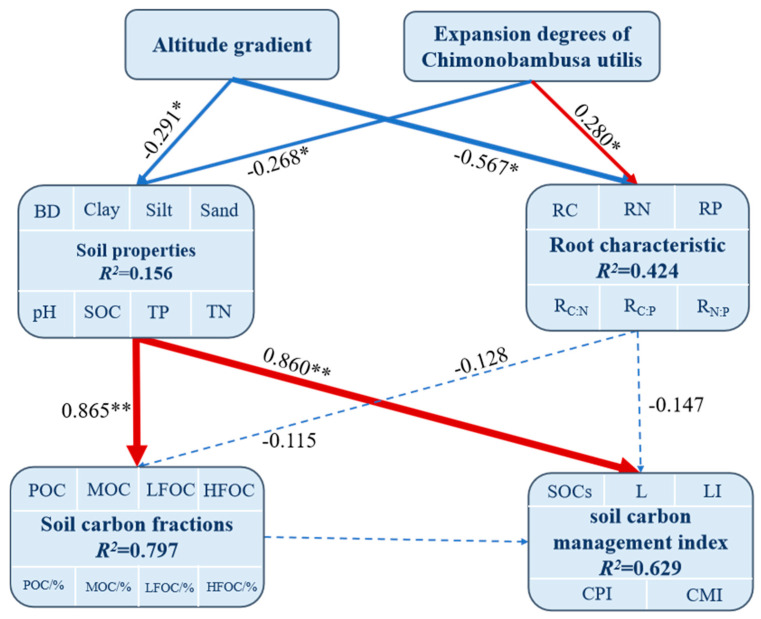
Structural equation modeling (SEM) analysis of the direct or indirect effects of root elemental composition, soil properties, altitudinal gradient, and expansion degrees of *Chimonobambusa utilis* on soil carbon fractions and the soil pool management index. Note: A total of 225 soil samples were collected across 5 altitudinal gradients, with 3 bamboo expansion forest plots per altitude, each containing 3 replicate plots and 5 soil samples per plot. Indicators for each forest area were based on composite samples from 3 replicate plots (5 soil samples each) to reduce sampling variability. Error bars show the standard error (*p* < 0.05, one-way ANOVA with Tukey’s test). Red thick lines indicate positive significant correlations, blue solid lines indicate negative significant correlations, and blue dotted lines represent non-significant negative correlations. The thickness of the lines reflects the level of statistical significance. “**” indicates extremely significant correlation (*p* < 0.01), and “*” indicates significant correlation (*p* < 0.05).

Furthermore, path analysis was conducted to examine the relationships among the soil carbon fractions, pool management indices, and key influencing factors, with a particular emphasis on the root elemental composition and soil properties (Figure 7). Path coefficients and coefficients of determination (R^2^) were estimated via 5000 bootstrap resamples, with significance levels indicated as * (*p* < 0.05) and ** (*p* < 0.001). Compared with the competing models, the model demonstrated adequate goodness-of-fit, with GFI = 0.92, CFI = 0.96, RMSEA = 0.048, and the lowest AIC value, indicating superior model parsimony and fit. The results revealed that changes in soil properties directly and significantly affect soil carbon fractions or the soil carbon pool management index but indirectly affect the soil carbon pool management index by influencing environmental factors such as the root elemental composition and soil carbon fractions. Among them, the soil carbon pool management indices were highly significantly positively correlated with the soil properties (with a path coefficient of 0.860 **) and soil carbon fractions (with a path coefficient of 0.450 **) and negatively correlated with the root elemental composition (with a path coefficient of 0.147). Specific indirect effects indicated that the altitudinal gradient and expansion degrees of *Chimonobambusa utilis* affected the root elemental composition, thereby altering the soil properties, promoting the transformation of soil carbon fractions and ultimately impacting the soil carbon pool management index, with this pathway showing a highly significant positive correlation (*p* < 0.001).

## 4. Discussion

### 4.1. Altitude Effects on Root-Soil C-N-P Stoichiometry Across Varying Expansion Degrees of Chimonobambusa utilis

Altitude is a critical factor affecting the content and stoichiometric ratios of carbon (C), nitrogen (N), and phosphorus (P) within the root-soil system of *Chimonobambusa utilis* in high-altitude karst mountainous regions. Fine roots serve as key components in the cycling of soil carbon and essential nutrients such as nitrogen and phosphorus. Some studies have shown that neglecting fine root biomass may lead to underestimations of soil organic matter and nutrient turnover rates ranging from 20% to 80% [12,44]. Our study revealed that the ratios of root total carbon (C), nitrogen (N), root carbon to phosphorus (R_C:P_), and root nitrogen to phosphorus (R_N:P_) generally decrease with increasing altitude, whereas the root carbon to nitrogen ratio (R_C:N_) tends to increase. This finding is consistent with the research results of [13], which may be attributed to several factors. First, the sudden decrease in temperature at high altitudes may limit the absorption of nutrients and water by *Chimonobambusa utilis*, resulting in greater translocation of photosynthetic resources from aboveground parts, such as leaves and culms, to underground components. Moreover, *Chimonobambusa utilis* may require increased phosphorus accumulation to sustain a relatively high growth rate in high-altitude cold environments, where the growing season is shorter than that in low-altitude regions. However, research by [5,45] on three plant species across three distinct altitudes in the Himalayas revealed that the N and P contents in plant roots decreased with increasing altitude, whereas the N:P ratio tended to increase. The observed discrepancy can be attributed primarily to two key factors [37,46,47]. First, there are interspecific variations in N and P nutrient requirements. Specifically, reference [3] focused on three deciduous tree species, whereas our research examined a monotypic species of *Chimonobambusa utilis*. Second, variations in soil conditions significantly influence nutrient content, as plant-derived chemical elements are largely derived from the soil, and their concentrations are closely correlated with the corresponding elemental composition of the soil matrix.

The nutrient composition of fine roots is closely linked to plant physiological processes and plays a crucial role in determining plant invasion potential. Previous studies have shown that during the encroachment of bamboo into broad-leaved forests, the increasing dominance of bamboo leads to a significant decrease in the contents of C, N, and P and the C:N ratio of fine roots [26,47]. This phenomenon can be attributed to the higher growth rate of bamboo in mixed bamboo and broad-leaved forests than in pure bamboo forests, which necessitates greater nitrogen and phosphorus concentrations and results in a reduced C:N ratio [15,22]. However, our study revealed that the concentrations of C, N, and P and the C:N ratio in the root system increased significantly with increasing expansion degree of *Chimonobambusa utilis* at high altitudes in the Jinfo karst mountains, which contrasts notably with the findings of previous studies. This is attributed primarily to the substantial resource limitations and environmental pressures in the high-altitude Jinfo karst mountain region. These conditions pose challenges for the efficient acquisition of N and P by *Chimonobambusa utilis*, thereby limiting its capacity to support rapid growth. As a result, *Chimonobambusa* utilizes fewer resources for N and P, leading to reduced N and P contents in fine roots. Moreover, as *Chimonobambusa utilis* gradually shifted from mixed forests to single-dominant communities, the spatial density of its fine roots increased [1,23]. Consequently, the demand for structural carbon increases to support the mechanical integrity of the expanding fine root system, resulting in a continuous increase in the fine root carbon content. This pattern led to a gradual decline in the soil C:N ratio, reflecting an adaptive strategy of *Chimonobambusa utilis* that prioritizes the accumulation of structural C to increase stress resistance while simultaneously conserving limited N and P resources to support survival. Consequently, our findings contrast markedly with those of previous studies on low-altitude Moso bamboo, which adopted a strategy characterized by rapid growth associated with low carbon content, high nitrogen and phosphorus contents, and a low C:N ratio.

### 4.2. Altitude Effects on Soil Organic Carbon Fractions and Pool Management Index Across Varying Expansion Degrees of Chimonobambusa utilis

The altitudinal gradient affects the distribution of water and heat in karst mountainous areas, which in turn influences vegetation patterns and soil physicochemical properties [37]. Some studies have demonstrated that the concentration of active organic carbon is significantly greater in high-altitude regions than in low-altitude regions [38,47]. However, we also found that the maximum values of POC, MOC, and LFOC occurred at the lower altitude range of 1300–1500 m, whereas the maximum value of HFOC was observed at the highest altitude range of 2100–2300 m. This occurred because the lower altitude range of 1300–1500 m has a suitable temperature and humidity, moderate rock weathering, and excellent soil bulk density, aggregate structure and porosity conditions, which promote the accumulation of MOC. Moreover, the soil aggregates can encapsulate and protect POC and LFOC. In contrast, at the highest altitude range of 2100–2300 m, the large diurnal temperature range and low overall temperature significantly reduce the decomposition rate of organic matter, hinder its transformation into active organic carbon, and delay the mineralization of organic carbon [3,44]. Additionally, HFOC has a stable structure and is strongly associated with soil clay and mineral phases, conferring high resistance to erosion. Under the dual mechanisms of erosion-induced selective removal and mineral-mediated protection, HFOC is preferentially retained, gradually accumulates over time, and ultimately becomes the dominant component of soil organic carbon within this altitude range. In addition, we found that the POC:MOC ratio reached its minimum value, whereas the LFOC:HFOC ratio reached its maximum at a moderate expansion degree of *Chimonobambusa utilis*. This phenomenon can be attributed to the inputs being continuous and diverse during moderate expansion of *Chimonobambusa utilis*, providing abundant fresh organic matter that readily adsorbs onto and forms complexes with soil minerals, thereby facilitating rapid replenishment of MOC. Moreover, the fine roots of bamboo and deep roots of broad-leaved trees induce mild soil disturbance, leading to the breakdown of large macroaggregates that physically protect POC. This disruption exposes POC to microbial decomposition, resulting in accelerated turnover and limiting its accumulation relative to MOC. Additionally, most organic inputs remain in an unhumified or semihumified state and are predominantly present as LFOC, whereas the formation of HFOC is constrained by delayed humification processes.

The soil carbon pool management index can systematically reflect variations in SOC, as it is jointly determined by the soil carbon pool index and the carbon pool activity index. Research on Tianmu Mountain has revealed that soil L and LI initially increase and subsequently decrease with increasing altitude, suggesting a corresponding initial increase followed by a decrease in the stability of the soil carbon pool [5,39], which is consistent with the results of our study. Moreover, our findings indicate that the highest concentrations of soil L and LI were observed within the altitudinal gradient of 1900–2100 m, whereas the lowest concentrations were recorded within the altitudinal gradient of 2100–2300 m. This phenomenon can be attributed to the favorable temperature and humidity conditions observed at altitudes between 1300 m and 2100 m. These environmental factors cause *Chimonobambusa utilis* forests to transition from a “semirapid growth” phase to a “conservative growth” phase, which is accompanied by an increase in fine root biomass and litter accumulation. When combined with the protective effects of minerals such as calcium carbonate present in karst soils, only a limited portion of active carbon is converted into stable HFOC. As a result, the overall activity of the carbon pool is enhanced. When the altitude exceeds 2100 m, extremely low temperatures cause the *Chimonobambusa utilis* forest to adopt a “completely conservative growth” strategy, characterized by reduced litterfall and restricted fine root growth. Moreover, underground water leakage common in karst soils results in relatively low humidity in high-altitude regions [14]. Consequently, the proportion of the active carbon pool diminishes, ultimately resulting in the lowest levels of carbon pool activity and activity index. Moreover, this study revealed that with the expansion of *Chimonobambusa utilis* forests, the soil carbon pool management index increased accordingly, indicating gradual stabilization of the soil carbon pool. This occurred because after the expansion of the *Chimonobambusa utilis* forest and the formation of a single dominant community, the proportion of stable carbon increased, whereas the proportion of active carbon decreased, and the ratio of the two significantly increased, directly driving up the CPMI. Additionally, the dense root system of *Chimonobambusa utilis* promotes soil aggregate formation through root interpenetration, which increases the clay content and physically isolates organic carbon within the aggregates, thereby further reducing the risk of fluctuations in the soil carbon pool.

### 4.3. Contribution of Variables to Soil Organic Carbon Fractions and Pool Management Index Across Varying Expansion Degrees of Chimonobambusa utilis

Our correlation analysis revealed that the altitudinal gradient was significantly positively associated with the MOC, whereas the clay content and degree of expansion of *Chimonobambusa utilis* contributed minimally to the soil carbon pool quality [30,36]. These findings indicate that, with increasing altitude, lower temperatures decelerate the decomposition of organic matter, whereas enhanced erosion leads to significant losses of labile carbon fractions (POC and LFOC). In contrast, MOC is more resistant to erosion because of its strong association with clay particles and iron-aluminum oxides, enabling its retention and gradual enrichment in soil [33,37]. Furthermore, karst soils are derived from carbonate rock weathering and are characterized by inherently low clay contents, which diminishes the role of clay in carbon stabilization [20,34]; consequently, the protective mechanism of organic carbon is predominantly governed by organomineral interactions rather than clay-mediated protection. However, the expansion of *Chimonobambusa utilis forests* has not overcome the environmental constraints imposed by altitude and temperature, and associated changes in carbon inputs remain limited [33,37]. As a result, both the clay content and bamboo expansion exert only minor influences on the soil carbon pool quality, leading to a prevailing pattern in which “altitude is the primary controlling factor, while the effects of clay and expansion are negligible.” Furthermore, we also reported that the soil BD increased significantly with decreasing altitude and was strongly negatively correlated with the soil carbon fraction and management indicators, which aligns with the findings of [10]. At high altitudes, low temperatures and high humidity inhibit soil compaction, whereas freeze-thaw cycles disrupt compact structural components in soil aggregates, resulting in a well-developed porous system. This reduces BD, lowers root penetration resistance, and facilitates root expansion of C. utilis as well as the transformation of labile carbon into stable forms such as MOC and HFOC. In contrast, warmer temperatures and enhanced evaporation at lower elevations reduce soil moisture, strengthening interparticle cohesion and promoting compaction and pore occlusion. These changes impair soil aeration, restrict root growth, favor methane emissions under anaerobic conditions, and compromise carbon pool stability. The karst-specific conditions of high bedrock exposure and shallow soils further amplify these altitudinal contrasts. A lower elevation, characterized by gentler slopes and more advanced bedrock weathering, results in the accumulation of sand and silt, leading to finer surface textures. Conversely, high-altitude sites feature extremely thin soils enriched in coarse rock fragments, yielding a more porous and less compact soil matrix. Together, these lithological and topographic influences drive pronounced differences in bulk density and the carbon management index along the altitudinal gradient.

Moreover, our structural equation modeling revealed critical drivers regulating the soil carbon pool management index at *Chimonobambusa utilis* in high-altitude karst mountainous areas, highlighting the altitudinal gradient, expansion degrees of *Chimonobambusa utilis*, root elemental composition, and soil properties as key factors controlling soil carbon fractions [12,21]. The results of this model indicate that the soil carbon pool management index in *Chimonobambusa utilis* at high altitudes is influenced primarily by soil properties, including soil physical structure, chemical properties (e.g., pH), and the soil carbon fraction. Soil physical properties provide a structural foundation for carbon sequestration, whereas soil chemical properties contribute to carbon stock stability through pH regulation. Additionally, recalcitrant organic carbon, particularly mineral-associated organic carbon, directly enhances the quality and resilience of the soil carbon stock. Furthermore, the CMI was significantly indirectly negatively correlated with the elemental composition of the root system. This occurred because the expansion ratio of *Chimonobambusa utilis* disrupts the balance of root system elements, thereby reducing the carbon fixation capacity and accelerating the mineralization and decomposition of active organic carbon (POC and LFOC). These changes indirectly diminish effective carbon inputs and impede the conversion of carbon into stable forms, leading to a significant negative correlation with the CMI. Therefore, although karst soils exhibit considerable potential for carbon sequestration through direct positive effects, the root adaptive strategies employed by *Chimonobambusa* to cope with poor nutrient environments indirectly limit the availability of organic carbon inputs, thus constraining the increase in the carbon pool management index (CMI). This underscores the trade-off between plant adaptation mechanisms and the soil carbon sequestration capacity in karst ecosystems.

### 4.4. Practical Applications and Future Research Prospects

In general, we also demonstrated that the quality of the soil carbon pool under *Chimonobambusa utilis* reached optimal levels during moderate expansion stages within the 1900–2100 m altitude range. It is recommended that this elevation band be designated as a coordinated development zone for *Chimonobambusa utilis* and native broad-leaved forests. To achieve ecological and economic synergy, *Chimonobambusa utilis* expansion should be maintained at a moderate level through the implementation of a mixed “*Chimonobambusa utilis* + broad-leaved forest” model, with native broad-leaved tree species preserved as companion stands. Leveraging the root-mediated organic carbon input from *Chimonobambusa utilis* while maintaining favorable soil carbon pool conditions can help sustain long-term ecosystem functionality. In contrast, at altitudes below 1900 m or above 2100 m, the expansion of *Chimonobambusa utilis* poses a risk of disrupting the soil carbon balance. Therefore, planting scales must be strictly controlled, and a conservation red line for broad-leaved forests should be established. All forms of forestland conversion in these zones must be prohibited, with priority given to the restoration of original broad-leaved forest vegetation and biodiversity conservation to preserve the integrity of the native soil carbon pool structure. We also wish to further clarify that this study is specifically confined to the Karst Forest Reserve, where the research findings are closely aligned with the local geographical setting, climatic regime, and vegetation composition. The proposed management approaches and strategies effectively address the conflict between the expansion of *Chimonobambusa utilis* and the conservation of broad-leaved forests, thereby contributing to ecosystem stability and long-term sustainability. However, in regions affected by severe soil disturbances or exhibiting markedly different climatic conditions, the applicability of these findings is likely to be substantially limited. In such cases, targeted follow-up research is necessary to refine the technical parameters and adapt management strategies before practical implementation. To control the expansion of bamboo forests, overcrowded *Chimonobambusa utilis* individuals should be selectively removed in invaded areas, while native broad-leaved tree species should be reintroduced simultaneously to establish a stratified mixed forest composed of tree, shrub, and herbaceous layers. This integrated management approach effectively suppresses excessive bamboo growth and enhances the ecological stability and resilience of forest ecosystems.

Additionally, a limitation of this study is the interpretation of microbial- and vegetation-driven aspects of the carbon cycle, which relies on established patterns from existing regional studies and is indirectly supported by variations in soil organic carbon fractions. The current study lacks direct measurements of microbial biomass, enzyme activity, vegetation cover, and related biotic indicators and therefore cannot quantify the linkages between microbial processes, vegetation characteristics, and soil carbon dynamics. As a result, the underlying mechanisms remain unclear and require further empirical validation through targeted microbiological assays and comprehensive vegetation surveys. To enhance the understanding of the soil carbon distribution in karst mountainous regions, future research should expand the sampling depth range, implement long-term monitoring, conduct geographically extensive evaluations under contrasting environmental conditions, and integrate additional ecological indicators to better characterize the spatiotemporal dynamics of soil carbon pools.

## 5. Conclusions

This study investigated soil organic carbon (SOC) fractions, stocks, and the carbon pool management index in *Chimonobambusa utilis*-invaded karst mountain ecosystems of Jinfo Mountain across an altitudinal gradient, with an assessment of key environmental drivers including altitude, expansion degree, root characteristics, and soil properties. The results revealed that (1) the root total carbon (TC) and total nitrogen (TN) contents increased significantly with increasing expansion degree and altitudinal gradient. (2) The particulate organic carbon (POC), TN, C:P, and N:P ratios reached their maximum values at the 1300–1500 m altitudinal gradient (45.75, 2.86, 22.96, and 25.60 g kg^−1^, respectively), and their minimum values occurred at 1900–2100 m (30.98, 0.87, 14.93, and 20.88 g kg^−1^, respectively). In addition, the POC:MOC (0.64) ratio reached its minimum value, whereas the LFOC:HFOC (1.63) ratio reached its maximum at a moderate expansion degree of *Chimonobambusa utilis*. In contrast, L (2.38), LI (2.01), and CMI (174.55) attained peak values at moderate expansion stages within the 1900–2100 m altitudinal gradient. The soil carbon pool management index increased with expansion degree, indicating progressive stabilization of the soil carbon pool. (3) Redundancy analysis revealed that the altitudinal gradient was significantly positively associated with the MOC content, whereas the clay content and degree of expansion of *Chimonobambusa utilis* contributed minimally to the soil carbon pool quality, whereas the BD was negatively correlated. Structural equation modeling demonstrated that the carbon pool management index was highly positively correlated with the soil properties (path coefficient: 0.860 **) and carbon fractions (path coefficient: 0.450 **) but negatively associated with the root elemental composition (path coefficient: 0.147). Overall, alleviating constraints on root-mediated organic carbon inputs across an altitudinal gradient may increase the adaptability of *Chimonobambusa utilis* to nutrient-poor environments. Furthermore, the soil carbon pool quality reached optimal levels during moderate expansion phases within the 1900–2100 m altitudinal gradient. The long-term dynamic monitoring of soil carbon pools should be enhanced, and the vegetation composition should be strategically optimized to improve the organic carbon input efficiency. These measures could contribute to increasing the carbon sequestration capacity and improving the carbon management index in high-altitude karst bamboo forest ecosystems.

## Data Availability

The data used in this study are confidential; however, all data can be made available upon reasonable request by contacting the corresponding author.
